# ALLSTAR: inference of reliAble causaL ruLes between Somatic muTAtions and canceR phenotypes

**DOI:** 10.1093/bioinformatics/btae449

**Published:** 2024-07-22

**Authors:** Dario Simionato, Antonio Collesei, Federica Miglietta, Fabio Vandin

**Affiliations:** Department of Information Engineering, University of Padua, Via Giovanni Gradenigo 6b, Padua, 35131, Italy; Department of Surgery, Oncology and Gastroenterology, University of Padua, Padua, 35128, Italy; Bioinformatics, Clinical Research Unit, Veneto Institute of Oncology IOV-IRCCS, Padua, 35128, Italy; Department of Surgery, Oncology and Gastroenterology, University of Padua, Padua, 35128, Italy; Oncology 2, Veneto Institute of Oncology IOV-IRCCS, Padua, 35128, Italy; Department of Information Engineering, University of Padua, Via Giovanni Gradenigo 6b, Padua, 35131, Italy

## Abstract

**Motivation:**

Recent advances in DNA sequencing technologies have allowed the detailed characterization of genomes in large cohorts of tumors, highlighting their extreme heterogeneity, with no two tumors sharing the same complement of somatic mutations. Such heterogeneity hinders our ability to identify somatic mutations important for the disease, including mutations that determine clinically relevant phenotypes (e.g. cancer subtypes). Several tools have been developed to identify somatic mutations related to cancer phenotypes. However, such tools identify correlations between somatic mutations and cancer phenotypes, with no guarantee of highlighting causal relations.

**Results:**

We describe ALLSTAR, a novel tool to infer reliable causal relations between somatic mutations and cancer phenotypes. ALLSTAR identifies reliable causal rules highlighting combinations of somatic mutations with the highest impact in terms of average effect on the phenotype. While we prove that the underlying computational problem is NP-hard, we develop a branch-and-bound approach that employs protein–protein interaction networks and novel bounds for pruning the search space, while properly correcting for multiple hypothesis testing. Our extensive experimental evaluation on synthetic data shows that our tool is able to identify reliable causal relations in large cancer cohorts. Moreover, the reliable causal rules identified by our tool in cancer data show that our approach identifies several somatic mutations known to be relevant for cancer phenotypes as well as novel biologically meaningful relations.

**Availability and implementation:**

Code, data, and scripts to reproduce the experiments available at https://github.com/VandinLab/ALLSTAR.

## 1 Introduction

In the last 10 years, the advances in DNA sequencing technologies have allowed to precisely depict the landscape of somatic alterations in large cohorts of tumors for various cancer types (Hudson *et al.* 2010, Weinstein *et al.* 2013, Mardis 2019). The study of these data has shown that cancer is characterized by an extreme inter-tumor *heterogeneity*, with the alterations observed in different tumors being almost entirely different for any pairs of tumors. A number of computational tools have been designed to try to identify the alterations that drive the insurgence and development of tumors, while tackling inter-tumor heterogeneity (Cortés-Ciriano *et al.* 2022). These tools are able to detect various types of signals (Vandin *et al.* 2012, Cibulskis *et al.* 2013, Mularoni *et al.* 2016, Arnedo-Pac *et al.* 2019) and integrate different prior and/or clinical information (Cowen *et al.* 2017, Sarto Basso *et al.* 2019, Reyna *et al.* 2020), but a common feature of such tools is that they detect alterations *correlated* with cancer phenotypes. That is, they identify alterations, or groups of alterations, significantly enriched in a group of patients or significantly associated with a (clinical) phenotype.

While the identification of alterations correlated with cancer phenotypes provides interesting insights into cancer initiation and progression, it does not guarantee that causal relations between somatic mutations and cancer are reported. While experimental and clinical validation is a necessary step to demonstrate the significance of alterations, tools reporting causal relations with guarantees on the quality of their findings would greatly reduce the resources needed to identify relevant alterations in follow-up experimental and clinical studies.

Randomized control trials are the gold standard in observational studies (Concato *et al.* 2000, Rosenbaum *et al.* 2010), and, in recent years, a lot of attention has been devoted towards mining *causal* rules (Silverstein *et al.* 2000) from observational data. Recently, Budhathoki *et al.* (2021) proposed a novel estimator of a rule’s effect, taking into account the uncertainty of the estimates derived from data, and developed a branch and bound algorithm for the discovery task. Similarly, our work aims at finding reliable causal rules, but properly implements a correction for controlling the Family-Wise Error Rate (FWER) in a multiple hypothesis testing scenario, which is a fundamental feature of cancer studies given the high number of alterations found in tumors.

In this article, we describe ALLSTAR, a novel tool to identify reliable causal relations between somatic mutations and cancer phenotypes. ALLSTAR identifies causal relations in the form of *rules* highlighting combinations of alterations with the highest average effect on the phenotype. Our contributions are 4-fold. Firstly, we prove that the underlying computational problem is NP-hard. Secondly, we show that one needs to properly correct for multiple hypothesis testing when identifying *reliable* causal rules. Thirdly, we design ALLSTAR, an effective branch-and-bound algorithm to identify the *k* rules with the highest reliable average effect on the phenotype, with guarantees on the FWER of the output. ALLSTAR identifies rules where genes are connected in a large interaction graph provided in input, and employs an iterative procedure leading to the identification of diverse rules, which highlight different causal relations potentially linked to cancer heterogeneity. Fourthly, we perform an extensive evaluation of ALLSTAR on both synthetic and cancer data, showing the statistical robustness of ALLSTAR and its ability to report well-supported as well as potentially novel causal relations between somatic mutations and cancer phenotypes.

ALLSTAR focuses on estimating the impact of genomic alterations on a tumor subtype, unlike Bayesian approaches such as Zhang *et al.* (2014) that learned a causal graph from The Cancer Genome Atlas (TCGA) mutation data to identify alterations relevant to ovarian cancer but without considering their effect on a target variable. A step towards the identification of causal relations between multi-omics data and a target variable (e.g. phenotype) has been made by the tools Aristotle (Mansouri *et al.* 2022) and CauMu (Liu *et al.* 2022), both identifying single features (i.e. alterations or genes) linked to the phenotype. Our tool provides an efficient approach to identify rules comprising multiple features, which is an important characteristic given the high inter-tumor heterogeneity. Moreover, Aristotle focuses on the significance of the relation (by computing a corresponding *P*-value), rather than their effect as done by ALLSTAR. Other causal tools, instead, leverage the increasing availability of single-cell RNAseq data (e.g. Cifuentes-Bernal *et al.* 2022) and the estimates of pseudo-time derived from such data to identify causal relations at the transcriptomic level.

## 2 Materials and methods

### 2.1 Causal rules

Causal rules study the influence that a subset of actionable variables $X=\{X_{1},\ldots ,X_{n}\}$ exert on a target variable *Y* accounting for the possible confounding influence of a set of control variables $Z=\{Z_{1},\ldots ,Z_{m}\}$, that are common causes of at least one $X_{i}\in X$ and *Y*. More specifically, a rule $\sigma =\pi_{1}\wedge \pi_{2}\wedge \ldots \wedge \pi_{\ell }$ is a conjunction of Boolean propositions *π_i_* defined on an actionable variable (e.g. $\pi_{i}\equiv X_{5}=1$), and which evaluates as *true* (⊤) under an assignment $x=\{x_{1},\ldots ,x_{n}\}$ if all its propositions are verified by setting each *X_j_* to the value *x_j_* (otherwise the rule *σ* is *false*, or ⊥). The *causal effect* of a rule *σ* (Budhathoki *et al.* 2021) on the target variable *Y* taking value *y* is defined as


$$e^{y}(\sigma )=\sum_{z}(p(Y|\sigma (x)=⊤,Z=z)-p(Y|\sigma (x)=⊥,Z=z))p(Z=z),$$

where σ(x) represents the value of *σ* under assignment **x**. $e^{y}(\sigma )$ takes value in [−1,1] and it measures the increase in the probability that the target *Y* takes value *y* when the rule *σ* is true w.r.t. when *σ* is false. Despite being defined on conditional probabilities, $e^{y}(\sigma )$ measures the *causal* influence that the variables composing the propositions in *σ* exert on the event *Y *=* y* if the *admissible input structure* assumptions (Budhathoki *et al.* 2021) are met, that are:

1. the target variable *Y* is not a cause of any $X_{i}\in X$;
2. none of the variables $X_{i}\in X$ is a cause of any $Z_{j}\in Z$;
3. none of the variables $X_{i}\in X$ is a cause of any other $X_{j}\in X$; and
4. there is no unobserved variable *U* that directly cause $X_{i}\in X$.

In other words, if the admissible input assumptions are met, $e^{y}(\sigma )$ measures the average treatment effect that the variables in *σ* exert on the event *Y* taking value *y* without including any spurious (i.e. non-causal) statistical correlation.

In this work we focus on applying the framework above to somatic mutations in cancer datasets, defining **X**, **Z**, and *Y* as follows:

1. the set **X** of features includes somatic alterations [i.e. Single Nucleotide Variants (SNV), loss of heterozygosity (LOH), hypermethylation] in a set of genes, and the observations are provided by a binary matrix describing the status (present or not) of such alterations in a cohort of patients;
2. the set **Z** of confounders includes relevant germline mutations and clinical information (i.e. race, age, etc.), and the observations are provided by a corresponding matrix of relevant clinical variables;
3. the target *Y* is a phenotype of interest, such as histological or molecular marker-derived cancer subtypes

In our setting, the constraints required by an admissible input structure for causal rule discovery translate as follows: 1. the target variable *Y* does not cause somatic alterations; 2. there is no somatic alteration that is a cause of any confounder; 3. there are no causal relations between somatic alterations; 4. there are no causal relations between somatic alterations and relevant unobserved variables. Assumptions 1, 2, and 4 are satisfied by a proper choice of target variable *Y*, of confounders **Z**, and the features **X** to include in the study. As for Assumption 3, note that we are considering here somatic alterations that are observed in the final tumor. Somatic alterations *arise* independently in the genome (even if some alterations, such as the ones affecting DNA repair, may alter the overall mutation rate); however, the fact that we are considering only somatic alterations observed in tumors that lead to an observation (i.e. were detected in a patient), means that the assumption of independence may not be satisfied in terms of *observed* alterations. When dependencies between alterations are known, one should remove them from the analysis, even if our experimental evaluation on synthetic data (Section 4.2) shows that ALLSTAR correctly identifies true causal rules when Assumption 3 is not satisfied, despite not providing formal guarantees on the results.

In such setup, each rule represents the observation of a specific set of gene alterations that occur simultaneously, and the rule effect is a measure of the influence of such pattern on having a specific cancer type.

### 2.2 Effect estimation from data

The causal effect is defined on probabilistic quantities that are usually unknown and should be estimated from a dataset D of observations on the variables X∪Z∪{Y}. Budhathoki *et al.* (2021) introduced the *reliable* estimator ${\hat{e}}_{\text{rel}}^{y}(\sigma )$, a biased estimator for $e^{y}(\sigma )$ with lower variance w.r.t. its estimation using only empirical probabilities, accounting for statistical noise using a confidence interval that contains the true rule effect with confidence 1−α, where α∈(0,1) is user-defined. Such estimator is correct for the effect estimation of just one rule, but it may lead to false positives if multiple hypotheses are analyzed, as in our case of discovering the top-*k* rules with the largest effect. As we focus on discovering rules bounding the FWER, that is the probability of returning in output at least one false positive, we correct the estimator proposed in Budhathoki *et al.* (2021) for multiple hypothesis testing using Bonferroni correction (Bonferroni 1936), i.e., we consider a corrected threshold $\alpha_{c}=\alpha /N$ for each hypothesis where *N* is the number of (potential) hypotheses tested.

## 3 ALLSTAR: inferring reliable causal rules between somatic mutations and cancer phenotypes

In this section, we present our algorithm ALLSTAR (reliable cAusaL ruLe discovery between Somatic muTations and cAnceR phenotypes) for causal rule discovery with guarantees on its results. The underlying problem of estimating the rule with highest causal effect is NP-hard even if the probability distributions are known *a priori* (the proof of NP-hardness is in the Supplemental Materials). ALLSTAR exploits a gene–gene interaction network to focus on sets of functionally related genes and prune the search space. The pseudocode of ALLSTAR is in Algorithm 1 (see the Supplemental Materials for a detailed explanation).


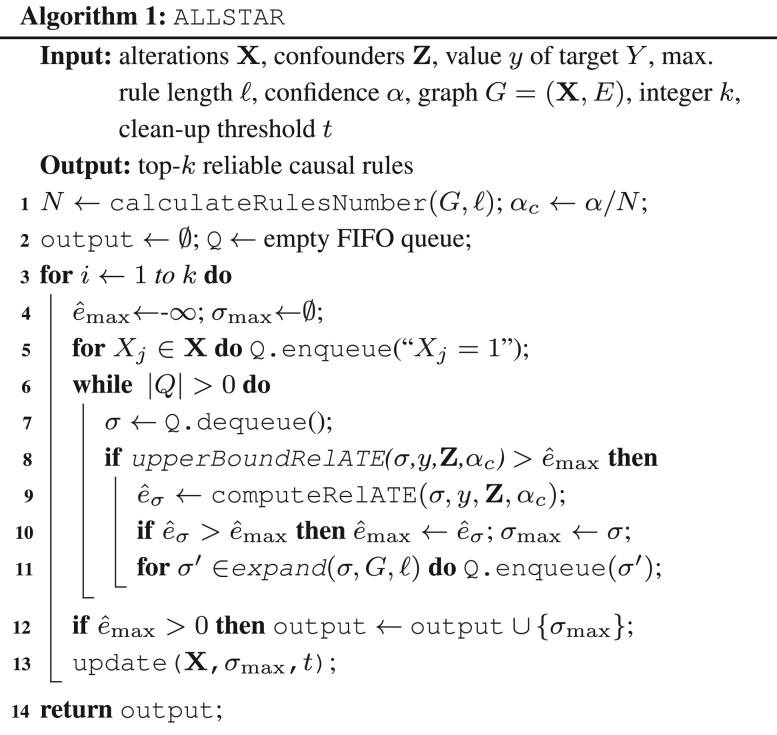


**Figure 1.**
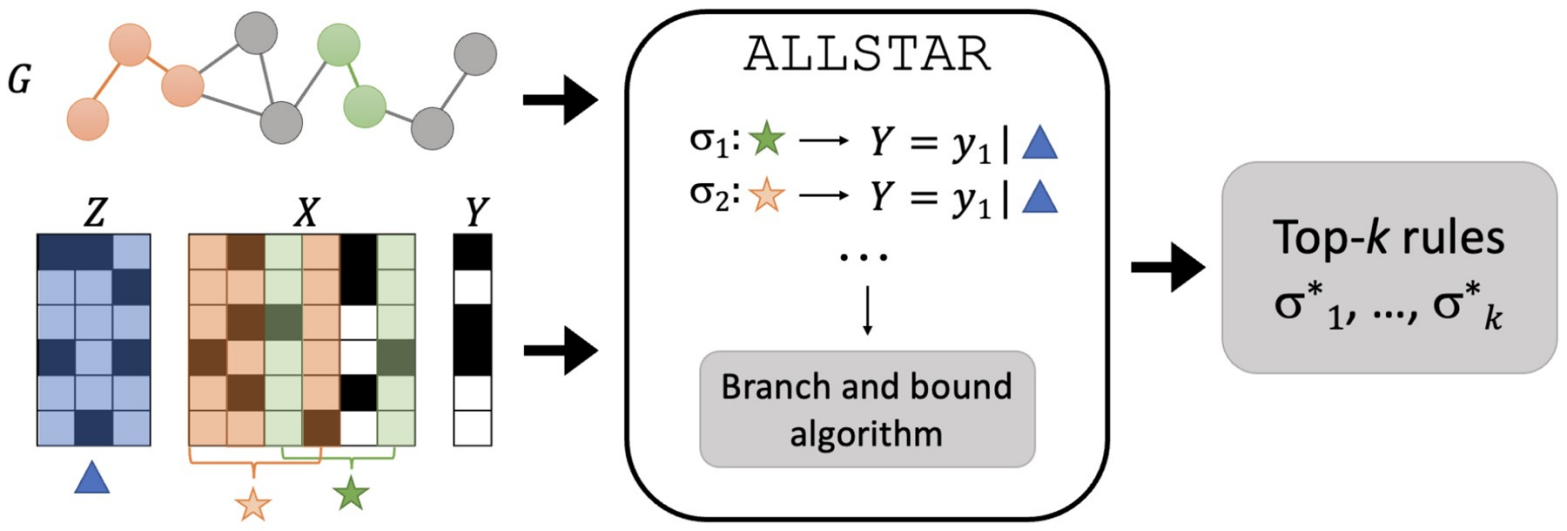
An illustration of ALLSTAR framework. From a dataset comprising a set of confounders **Z**, treatments **X**, and a target *Y*, ALLSTAR uses a branch and bound approach to discover the top-*k* rules $\sigma_{1}^{*},\ldots ,\sigma_{k}^{*}$ with the highest reliable causal effect. ALLSTAR exploits a gene–gene interaction network *G* to focus on biologically meaningful rules.

At its core, ALLSTAR (see Fig. 1) employs the branch-and-bound approach proposed in Budhathoki *et al.* (2021) to discover the rule with the highest causal effect, while limiting to rules with at most ℓ alterations. Moreover, since in practice we are interested in finding multiple and diverse rules with positive reliable effect and with functionally related alterations, ALLSTAR uses an iterative approach to identify at most *k* rules, where *k* is a parameter provided by the user, and an interaction graph *G* to consider only rules with functionally related alterations.

Specifically, ALLSTAR takes in input a set **X** of alterations, a set **Z** of confounders, a value *y* of interest for the target variable *Y*, the maximum length ℓ of rules, a confidence level *α*, a graph *G* whose vertices are the alterations in **X** and whose edges represent some relation between alterations (e.g. an edge represents the interaction between the proteins where the alterations are found), the maximum number *k* of rules to be reported in output, and a clean-up threshold t∈[0,1] that controls the diversity of the rules reported in output. In output, ALLSTAR produces at most *k* rules containing up to ℓ alterations, with the highest reliable effect and where each rule consists of alterations that form a connected subgraph of *G*. In addition, each reported rule comprises alterations that appear in a set of patients different from the alterations in other reported rules, where the difference is controlled by the parameter *t*.

The following theorem proves that ALLSTAR produces in output a set of rules with a rigorous bound on its FWER, where a false positive is defined as a rule *σ* reported in output but with effect e(σ)≤0. (The proof is in the Supplemental Materials.)

Theorem 1. *ALLSTAR* (**X**, **Z**, *y*, ℓ, *α*, G=(X,E), *k*, *t*) outputs a set of rules with FWER≤α.

While the parallel implementation of ALLSTAR employs the branch-and-bound approach proposed in Budhathoki *et al.* (2021), we also develop an improved (i.e. tighter) upper bound on the reliable causal effect of a rule that is best suited for single-core runs since it requires a data structure shared among cores. Such bound relies on the key observation that one rule σ′ is more specific of every rule in $\Omega_{p}=\{\sigma \prime \setminus \{\wedge \pi_{k}\}|\forall \pi_{k}\in \sigma \prime \}$ (Details in the Supplemental Materials).

As stated above, in order to identify a diverse and more informative set of rules, the set **X** of alterations is updated after each rule is extracted. This is done with function update(**X**,$\sigma_{\max}$,*t*) (line 13), which we now describe. Such function removes from the set of alterations **X** the ones that either appear in the rule $\sigma_{\max}$ or are very similar to at least one alteration in $\sigma_{\max}$. The similarity is defined according to the normalized city-block Manhattan distance, defined for two vectors **a** and **b** in *n* dimensions as $d_{M}\left(a,b\right)=\frac{1}{n}\sum_{i=1}^{n}\left|a_{i}-b_{i}\right|$. In particular, update(**X**, $\sigma_{\max}$, *t*) removes from **X** all alterations in $\sigma_{\max}$ and the ones with distance *d_M_* less than *t* from at least one alteration in $\sigma_{\max}$, where the distance between the vectors describing the appearance of alterations in patients is considered and *t* is a user-defined threshold. This function therefore allows to recover non-overlapping rules over the whole alterations’ search space.

We implemented ALLSTAR in Python 3. Our implementation exploits multicore parallelism, when available. Code, data, and scripts to reproduce the experiments described below are available at https://github.com/VandinLab/ALLSTAR.

## 4 Experimental evaluation

This section describes the experimental evaluation of our algorithm ALLSTAR on synthetic and cancer datasets. Section 4.1 describes the cancer data we used. Section 4.2 presents the results on synthetic data, and, finally, Section 4.3 describes the results on breast cancer data.

### 4.1 Cancer data and interaction network

We tested ALLSTAR on publicly available breast cancer (BRCA) data from TCGA. In particular, we downloaded public clinical and somatic mutational data from the TCGA-BRCA repository, for a total of 1096 samples. We also included the subtype classification of TCGA-BRCA based on the 50-gene PAM50 model (Parker *et al.* 2009). We also retrieved germline mutational patterns for TCGA patients in BRCA1 and BRCA2 from Kraya *et al.* (2019). We integrated two additional alteration types that play a significant role in cancer: LOH information from Riaz *et al.* (2017) and reported by Bodily *et al.* (2020), and hypermethylation from Xena Functional Genomics Explorer data (Goldman *et al.* 2015) and reported in Bodily *et al.* (2020). The final datasets comprised a number of samples ranging from 898 to 935, depending on the target variable of interest. As an input graph for ALLSTAR we considered the most recent Functional Interaction (Wu *et al.* 2010) gene network from Reactome [FIsInGene (version 2021), available at this link.], which comprises almost 14 000 genes and more than 250 000 edges.

### 4.2 Results on synthetic data

In this section, we assess ALLSTAR’s performance on synthetic data. In particular, we preliminarily compare ALLSTAR with standard correlational approaches (We could not compare with Aristotle due to issues with its implementation, available at this link.). We then use synthetic datasets to evaluate the impact of the multiple hypothesis testing correction employed by ALLSTAR and the effectiveness of using graph *G* to reduce the number of rules to evaluate. Lastly, we evaluate the ability of ALLSTAR to recover rules, involving multiple alterations, planted in a large, noisy dataset. We also assessed the stability of our algorithm on different combinations of the user-defined parameters ℓ, *G*, and *t*. Results highlight how increasing the number of combinations decreases the recovered effect without leading to any false discovery, and how ALLSTAR is stable w.r.t. Assumption 3 violations (see Section 2.1) under suitable settings of *t*. Such study as well as a performance analysis highlighting the equivalence or advantage (depending on the rule length) of a branch-and-bound approach over a brute force approach are shown in Section 3 of the Supplemental Material.

**Figure 2.**
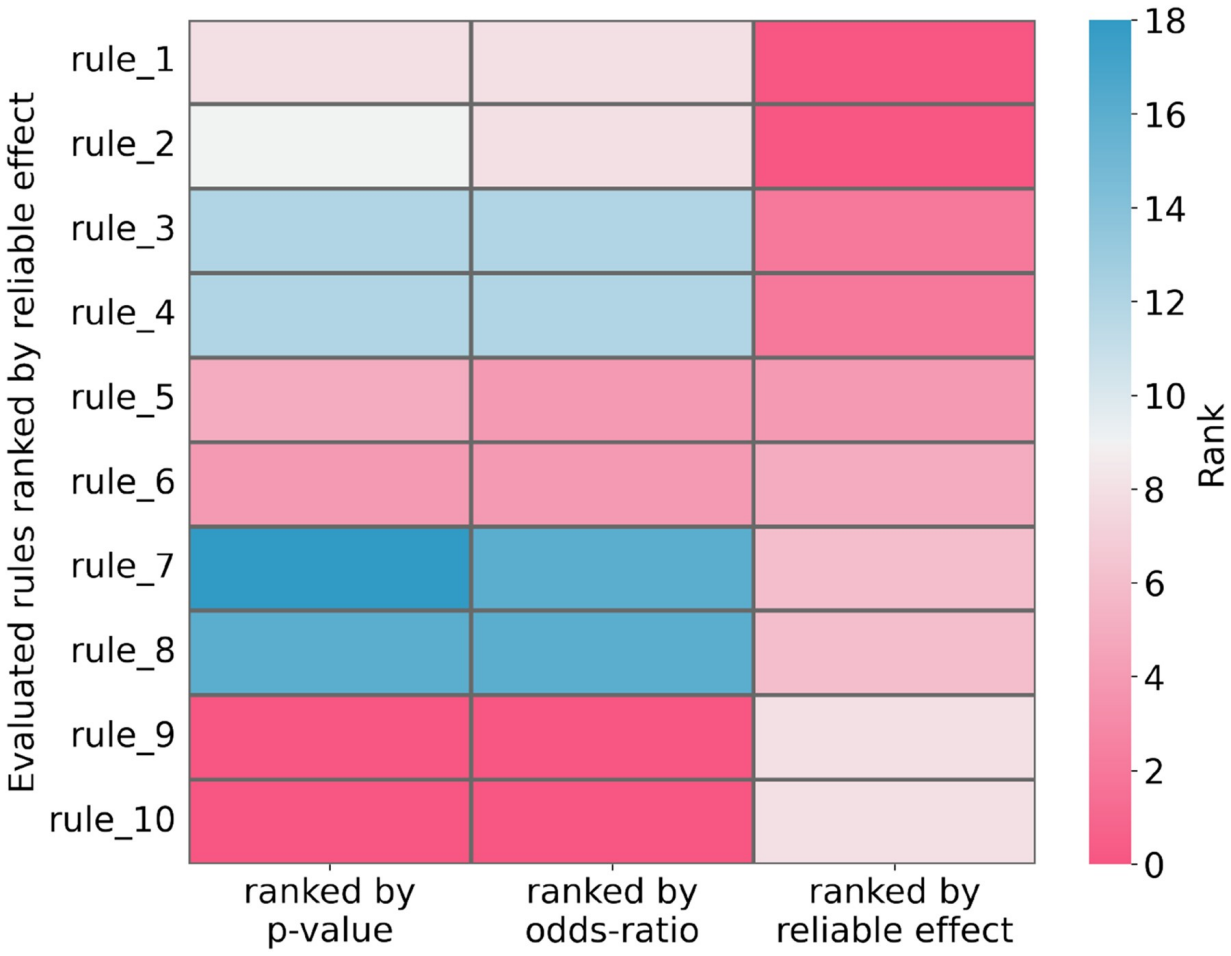
Ranking comparison of the top-10 binary rules with the highest effect computed on a dataset of 100 samples w.r.t. three different metrics (*P*-value, odds-ratio, and reliable effect). Each row corresponds to a rule and each column corresponds to its ranking (with first scores being the highest) w.r.t. *P*-value, odds-ratio, and ALLSTAR reliable effect, respectively.

In the preliminary experiment, we compared ALLSTAR with standard correlational approaches, to understand whether the results obtained are the same. In particular, we generated all possible binary rules comprising one gene and one target on a dataset of 100 samples, and for each one we computed (i) the reliable effect as computed by ALLSTAR, (ii) the *P*-value from the Fisher exact test, and (iii) the odds ratio (In the two latter cases, we computed the values taking into consideration the contingency table associated to each rule as in Budhathoki *et al.* 2021.). We then sorted the results according to each computed value and compared the three rankings. In Fig. 2 we show the rankings of the top-10 rules with the highest reliable effect: the top rules obtained by ALLSTAR have a much lower ranking as if they were ranked by *P*-value or odds ratio. For example, 4 of the top-10 rules according to the reliable effect are not in the top-10 by *P*-value or by odds ratio, with one rule appearing in the 18th position of the ranking by *P*-value. In general, while there is a concordance in terms of Kendall-tau coefficient (Kendall 1938) between the ranking by reliable effect and the other measures (Kendall-tau coefficient has 0.79 correlation between the odds ratio and effects, and 0.9 between *P*-values and effects), the reliable effect provides different top rules (which are the most interesting ones for any practical purpose) than standard correlation approaches. An even larger difference between rankings appears in real-world cancer data (see Section 5 in the Supplemental Material).

We then performed three experiments to assess ALLSTAR’s efficiency and correctness, using synthetic datasets. Every synthetic dataset resembles the structure of real cancer data, with mutated genes as treatments **X** and a binary outcome *Y*. (For simplicity we set Z=∅ in these analyses.) For each experiment, we sampled 10 datasets for every tested sample size (25, 50, 75, 100, 250, 500, 1000, 5000, 10 000, and 25 000). In each dataset, most alterations are drawn randomly with probability 0.5 and independently of the outcome *Y*. In some datasets, we planted alterations with a causal relation to the target *Y*; such alterations constitute the rules of interest to assess ALLSTAR’s performance. Their relationships with *Y* are described in the related paragraph below.

In the first experiment, we assessed the impact of correcting for multiple hypothesis testing on false positives. In this experiment we considered only random alterations in each sample, hence, no causal rule (i.e. any rule with a positive effect) with respect to the outcome was planted. We considered three different estimates of the (reliable) effect: the version based on the naïve estimate of probabilities, the reliable approach proposed in Budhathoki *et al.* (2021) (The code available at the bitbucket repository does not run properly, therefore we implemented our own, equivalent, version.), and the one used by ALLSTAR (see Section 2.2). For the last two estimates, the value α=0.05 was considered. In particular, the naïve approach estimates the effect $\hat{e}(\sigma )$ as the difference $\hat{p}(Y=y|\sigma =⊤)-\hat{p}(Y=y|\sigma =⊥)$ (i.e. empirical probabilities estimated from data and without any correction), while the reliable approach proposed in Budhathoki *et al.* (2021) considers ${\hat{e}}_{\text{rel}}(\sigma )$ (i.e. adding confidence bounds) but without correcting for multiple hypothesis testing, as it is done instead in our approach (see the Supplemental Materials for a detailed explanation).

Our findings show that both the naïve and reliable approach incorrectly return at least one rule with a positive effect for *every* dataset (i.e. corresponding to a FWER of 1), while ALLSTAR is the only one correctly returning zero false positives. These results show that the multiple hypothesis correction on ALLSTAR’s reliable effect is a crucial component to avoid false discoveries.

The second experiment assesses the effectiveness of using the interaction graph *G* in ALLSTAR when identifying causal rules composed of multiple alterations. We sampled multiple datasets with a total of 22 alterations, of which 7 are part of a rule causally related to the target *Y* and constitute a connected subgraph of *G*.

We ran ALLSTAR for various values of the maximum rule length ℓ. As expected, the estimate of the effect converges to the true effect for all values of ℓ, and the estimate obtained using the interaction graph *G* is significantly better than the one when no prior knowledge is considered. Moreover, the use of *G* drastically reduces the runtime (due to a reduction in the number of candidate rules). For example, with 25 000 samples and ℓ=7, the runtime using *G* is of few seconds, while almost 3 minutes are required when no prior knowledge is considered. This shows that the interaction graph leads to significant improvements in terms of the estimate of the true effect and of runtime.

Lastly, we ran an experiment to assess the ability of ALLSTAR to recover planted rules that cover a wide spectrum of diverse functional processes, a key feature given the high inter-tumor heterogeneity that characterizes cancer, even when some admissible input structure assumptions (see Section 2.1) are not satisfied. We simulated datasets with 3 planted rules (of five genes in total) and 100 random alterations. For each alteration in a planted rule, we also planted a correlated alteration with 97.5% of values identical to the planted alteration. This allows us to assess whether ALLSTAR reports the correct causal alterations and not the correlated (but not causal) ones. We obtained the top-three rules running both a variant of ALLSTAR obtained without using the Manhattan distance-based updating procedure (see function update(****X****,$\sigma_{\max}$,**t**) in Algorithm 1), and ALLSTAR with *t *=* *0.05. ALLSTAR reports the planted rules and correctly disregards the rules comprising the correlated alterations. The variant of ALLSTAR that does not use the Manhattan distance-based updating procedure, instead, produces, among the top-three rules, rules containing the correlated alterations. In the latter case, ALLSTAR returning correlations instead of causal relations is a consequence of the failure of Assumption 3 (more on that in the Supplemental Material) for the admissible causal structure, while the Manhattan distance-based updating procedure allowed us to remove the spuriously-linked variables and to report only causal relations, even if the causal effect may not be precisely estimated due to not all assumptions being satisfied. These results show that the use of the Manhattan distance-based updating procedure is important to focus on causal alterations and disregard alterations that are not causal but only correlated to causal ones.

### 4.3 Results on breast cancer data

In this section we provide more details on how we built the datasets, the parameters we used in our analyses with ALLSTAR, the results we obtained, and their biological relevance. We ran ALLSTAR on breast cancer data described in Section 4.1, split into treatments **X**, confounders **Z**, and outcomes *Y*. In each run, we considered a common set of confounders, while **X** and *Y* are combined in different ways in each run to focus on certain cancer mechanisms. In particular:

- We considered seven confounder variables **Z**: gender, race, age at diagnosis, menopause status, history of another previous malignancy, and the presence of a germline mutation in genes BRCA1 and BRCA2.
- We selected a total of 622 alterations, which include the 300 most frequently somatically mutated genes, the 300 most frequent LOHs, and 22 frequently hypermethylated genes. We performed two types of analyses: one where each alteration corresponds to a treatment (element of **X**), and one where we considered as treatments **X** the 300 most frequently altered genes by considering a gene mutated if any of the three alterations above is present.
- As target *Y*, we considered three sub-typing classifications: an histological categorization (Ductal, Lobular, and Other carcinoma), an expanded molecular one, based on gene expression (Basal, HER2E, Luminal-A, Luminal-B, and Normal-like), and a specific binary molecular classification (Triple-Negative, or not).

Since we considered cancer sub-type as target *Y*, and causal relations among somatic alterations are not known in this context, we did not remove alterations in our analyses. We tested ALLSTAR under multiple settings on the differently combined datasets: we set the maximum rule length ℓ from 2 to 4, *k *=* *3, and *t *=* *0.01. Data requirements increase exponentially with the size of **Z**, and, therefore, for each dataset we run ALLSTAR multiple times each time passing a different subset of **Z** of cardinality at most 1. On each run, we set α=0.05/(|Z|+1) to bound the FWER of all the tests on the same dataset below 0.05. Finally, we took into consideration both the presence and the absence of treatment. Table 1 shows the best rules with no confounders, at the top, and when conditioning on confounders, at the bottom. Note that patients in our dataset are all affected by cancer, therefore every reported rule implicitly conditions on such event.

**Table 1.** Best rules, with (bottom) and without (top) confounder’s conditioning, ordered by descending effect.

ID	Rule	Effect
*a*	CDH1_*som*_ = 1 → Lobular	0.470
*b*	CDH1_*som*_ = 1 ∧ ANK2_*som*_ = 0 ∧ SCN5A_*som*_ = 0 → Lobular	0.430
*c*	CDH1_*som*_ = 0 → Ductal carcinoma	0.401
*d*	ITGB3_*alt*_ = 1 ∧ RHOA_*alt*_ = 1 ∧ MAP3K1_*alt*_ = 1 → Basal	0.342
*e*	TP53_*som*_ = 1 ∧ ATRIP_*loh*_ = 1 ∧ ERBB2_*loh*_ = 1 → Basal	0.300
*f*	ITGB3_*alt*_ = 1 ∧ MAP3K1_*alt*_ = 1 → Basal	0.297
*g*	TP53_*som*_ = 0 → Luminal-A	0.289
*h*	RB1_*loh*_ = 1 ∧ PHB_*loh*_ = 1 ∧ LIMD1_*loh*_ = 1 → Basal	0.271
*i*	TP53_*som*_ = 0 ∧ BRCA1_*meth*_ = 0 → Luminal-A	0.268
*j*	ERBB2_*alt*_ = 1 ∧ MST1_*alt*_ = 1 → Basal	0.245
*k*	MST1_*loh*_ = 1 ∧ ERBB2_*loh*_ = 1 → Basal	0.243
*l*	RB1_*loh*_ = 1 ∧ PHB_*loh*_ = 1 → Basal	0.242
*m*	STAT3_*alt*_ = 1 ∧ ERBB2_*alt*_ = 1 ∧ WNT5A_*alt*_ = 1 → Basal	0.241
*n*	TP53_*alt*_ = 1 ∧ RB1_*alt*_ = 1 ∧ NGFR_*alt*_ = 1 → Basal	0.241
*o*	PIK3CA_*som*_ = 0 ∧ RHOA_*loh*_ = 1 ∧ NGFR_*loh*_ = 1 → Basal	0.240
*p*	TP53_*som*_ = 1 ∧ NME1_*loh*_ = 1 → Basal	0.230
*q*	TP53_*loh*_ = 1 ∧ PRKCD_*loh*_ = 1 ∧ NME1_*loh*_ = 1 → Basal	0.226
*r*	PDX1_*alt*_ = 1 ∧ SPOP_*alt*_ = 1 → Basal	0.203
*s*	TP53_*som*_ = 1 ∧ ERBB2_*loh*_ = 1 ∧ PRKCD_*loh*_ = 1 → TripleN	0.195
*t*	ITGB3_*alt*_ = 1 ∧ RHOA_*alt*_ = 1 ∧ MAP3K1_*alt*_ = 1 → TripleN	0.184
*u*	TP53_*som*_ = 0 ∧ BRCA2_*som*_ = 0 → Luminal-A | gender	0.243
*v*	CDH1_*som*_ = 1 ∧ AKT1_*som*_ = 0 → Lobular | age_at_diagnosis	0.229
*w*	ERBB2_*alt*_ = 1 ∧ RHOA_*alt*_ = 1 → Basal | BRCA2_*germ*_	0.202
*x*	TP53_*som*_ = 0 ∧ BRCA2_*som*_ = 0 ∧ BRCA1_*meth*_ = 0 → Luminal-A | gender	0.197
*y*	TP53_*som*_ = 0 ∧ RB1_*som*_ = 0 ∧ BRCA1_*meth*_ = 0 → Luminal-A | BRCA2_*germ*_	0.191
*z*	TP53_*som*_ = 1 ∧ ERBB2_*loh*_ = 1 → Basal | history_other_malignancy	0.175

Rules’ description is as follows: GENE1_alterationtype_ = [0,1] GENE2_alterationtype_ = [0,1] … → Target subtype | Confounder.

The first three rules by effect include gene CDH1, which is a recurrently mutated gene in breast cancer and whose impact has been recognized as substantial in lobular histological subtype (Erber and Hartmann 2020, McCart Reed *et al.* 2021), consistent with rules *a* and *b*; consistently, rule *c* states that the absence of an alteration in CDH1, given a breast cancer diagnosis, increases the chances of developing a ductal subtype, antagonist to the lobular one. Moreover, the combination of mutated CDH1 with unaltered ANK2 and SCN5A (rule *b*) provides an additional perspective on the mechanisms regulating the lobular subtype: ANK2 is typically downregulated in breast cancer, while SCN5A is upregulated in almost every neoplastic process. However, SCN5A is known to mediate the epithelial–mesenchymal transition (EMT), a biological trait underpinning cancer aggressiveness: the absence of a mutation in this gene can be interpreted as a normal state for EMT, aligned with the mild characteristics of the lobular subtype (Gradek *et al.* 2019, Luo *et al.* 2020). Additionally, the rule including the absence of mutation in AKT1 in lobular carcinoma (rule *v*) is coherent, since this gene is strongly associated with ductal differentiation (Hinz and Jücker 2019). As a plus, this rule is strengthened by the conditioning on the confounder “age at diagnosis,” which removes spurious correlations.

When considering a gene altered in the presence of either a somatic, LOH or hypermethylation, strong effects are linked to the molecular basal-like subtype. ALLSTAR reports the combination of aberrations occurring in ITGB3 and MAP3K (rule *f*) as strongly causal of the aforementioned subtype, in agreement with literature: Fuentes *et al.* (2020) and Li *et al.* (2022) converge on this conclusion due to their cancer-promoting activity and inclusion in the metastatic process. ITGB3 and MAP3K have recently gained attention relatively to basal-like breast cancer, but their combination is yet to be investigated. Even more interesting is the extension of this causal rule with the alteration of RHOA (rule *d*): the higher score of this expanded rule in association with basal-like subtype can be explained by the association of the outcome with precocious metastasization in accordance to RHOA’s anti-metastatic function (Kalpana *et al.* 2021).

Even more specific mechanisms are retrieved by considering the decomposed treatments. Besides the strong positive effect of mutated TP53, which is well ascertained in non-luminal breast cancer (Abubakar *et al.* 2019), even more relevant is the causal effect increase in combination with the LOH event in ATRIP (rule *e*). When stable, this gene is responsible for anti-proliferative signal mediation (Venere *et al.* 2007), but its impairment’s effect is not well established in the literature. The interaction with mutated TP53 is interesting but it needs further investigation.

Another combination strongly rooted in literature is LOH in RB1 and PHB (rule *l*), as explained by Wang *et al.* (1999): RB1 is an important tumor suppressor gene (Herschkowitz *et al.* 2008), while PHB mediates anti-proliferation signaling (Sato *et al.* 1992, Jupe *et al.* 1996), therefore their combined action, if altered, is easily explainable in basal-like tumors. The addition of LOH in LIMD1 (rule *h*) is less established in breast cancer, being more associated with lung carcinoma, but its oncosuppressive role, and the correlation between LOH and mitosis, make it a potential key player in basal subtype (Huggins and Andrulis 2008).

HER2-positive, basal-like, and triple-negative breast cancer are consistently determined by aberrations occurring in MST1 (Jin *et al.* 2021). Our findings (rules *j*, *k*) coherently overlap this knowledge, extending it by pairing MST1 and ERBB2 within the same positively-scored rule. ERBB2 is a member of the epidermal growth factor (EGF) receptor family and its overexpression in 20%–30% of invasive breast carcinomas leads to increased chemoresistance to certain chemotherapeutic agents (Tan and Yu 2007). Its mutational impact is undefined in literature, as only ERBB2’s expression abnormalities have been encountered in breast malignancies, especially in triple-negative/basal-like. Our result in this particular case is partially coherent but can enable further studies into the MST1–ERBB2 interaction in terms of mammalian carcinoma profiling. Conversely, the joint action between ERBB2, STAT3, and WNT5A (rule *m*) is more explainable. STAT3 has a pivotal role in the initiation, progression, metastasis, and immune evasion of triple-negative breast cancer (Qin *et al.* 2019), while WNT5A reduces the clonogenicity, invasiveness, migration, and proliferation of carcinoma cells, and it is also considered a therapeutic target (Kobayashi *et al.* 2018). The rule that ALLSTAR returned is not specific, as it emerged from the aggregated dataset, but it suggests a strong mutational involvement of these three genes in basal breast cancer. This being said, ERBB2 is recalled in rule *w* with RHOA: both genes offer potential reasons to be partnering in the determination of Basal subtype, but there is no clinical evidence of their combination, let alone an involvement of BRCA2 germline mutation as a confounder to condition over. This rule is a clear example of potential relations that need to be evaluated in future studies. It is not a surprise that various rules with a high conditional effect, which is one of the main contributions of choosing a causal approach, are related to one of the most debated genes, ERBB2, suggesting its direct involvement in breast cancer carcinogenesis (see also rule *z* in combination with TP53).

An additional point favorable to our methodology consists in the rules we have *not* found: long genes, such as TTN, HMCN1, or DMD, usually harbor several mutations simply due to their size. ALLSTAR seems robust to this drawback, even if those genes are in the top-20 of the most somatically mutated ones in TCGA data. As a term of comparison, Saravia *et al.* (2019) perform a chi-square test to detect meaningful mutations in triple-negative breast cancer, identifying TTN, HMCN1, and DMD, among others, as statistically significant players in recurrent patterns of genomic alterations with a potential contribution to tumor evolution. The authors themselves acknowledge the possibility their findings may be false positives and our results support this hypothesis.

As a further functional evaluation, we considered, for each analysis, the set of genes obtained by merging the alterations reported in any of the rules from ALLSTAR, and performed pathway enrichment analysis with DAVID (Huang *et al.* 2009) to find statistically overrepresented biological functions (encoded in KEGG database, Kanehisa and Goto 2000) in each of these sets of genes. We selected 0.05 as the significance cut-off for pathways’ *P*-value. We then counted the occurrence of each pathway, when significantly enriched, over all the results of the analyses we ran. The most represented pathway, occurring in 65% of the sets, is neurotrophin signaling pathway, whose relevance as a potential therapeutic target for breast cancer has been previously ascertained in preclinical studies (Hondermarck 2012). Interestingly, the breast cancer pathway (*KEGG: hsa05224*) occupies one of the top spots with a 57% of occurrence, alongside other known relevant pathways such as Rap1 and PI3K-Akt signaling (Zhang *et al.* 2017). Additionally, the fluid shear stress and atherosclerosis (*KEGG: hsa05418*) scored an occurrence of 60%: the impact of this process in breast cancer, and in oncogenesis in general, is still unclear. However, this result seems to endorse some preliminary findings: according to Choi *et al.* (2019), in addition to promoting hematopoietic growth, biomechanical forces seem to be significant microenvironmental variables in the generation of cancer stem-like cells (CSLCs) or tumor-initiating cells (TICs) in cancer metastasis.

## 5 Conclusions

We introduced ALLSTAR, a novel tool to identify causal relations between somatic alterations and cancer phenotypes from mutational data measured in large cohorts of cancer patients, in contrast to previous approaches focusing on correlations. Our tool reports rules defined on several interactions and integrates prior information in the form of a graph to focus on functionally related alterations. It also uses an iterative procedure to identify diverse rules to tackle inter-tumor heterogeneity. Our extensive experimental evaluation shows that ALLSTAR is an efficient and effective tool and that it identifies well-supported causal relations from cancer data.

## Supplementary Material

btae449_Supplementary_Data
