# Supplementary material for: ALLSTAR: inference of reliAble causaL ruLes between Somatic muTAtions and canceR phenotypes
Source: Bioinformatics. 2024 Jul 22;40(7):btae449. doi: 10.1093/bioinformatics/btae449 (PMC11520414; doi:10.1093/bioinformatics/btae449)
Supplement: btae449_Supplementary_Data [file btae449_supplementary_data.pdf]

Supplemental Material of ALLSTAR: Inference of ReliAble CausaL RuLes between Somatic MuTAtions and CanceR Phenotypes

1 Computational Problem Definition and NP-Hardness

We now define the computational problem at the core of finding causal rules. In particular, we consider the problem of finding the rule with the largest positive effect on a target variable, defined as follows.

Definition 1. ***Max Positive CRD problem.*** *Consider variables* $\mathbf{Z} \cup \mathbf{X}$ *and a target variable* Y*. Find the rule* σ^* *with i)* $e(\sigma^*) > 0$ *and ii)* $\sigma^* = \arg\max_\sigma e(\sigma)$.

The *Max Positive CRD* problem is a simplified version of the problem of finding the rule with largest positive effect from data, since it assumes that one has access to the *exact* probabilities for the events of interests, while, in practice, such probabilities are estimated from an observational dataset (see Section 2). Nonetheless, we prove that the problem above is computationally difficult. In particular, we prove that finding the causal rule with the maximum effect is NP-hard, even when no confounder is considered (i.e., when $\mathbf{Z} = \emptyset$) and the true probabilities are described by a Bayesian Network, that is a convienent mathematical way to represent causal relations between variables. Formally, Bayesian network (BN) is defined as a tuple $< \mathcal{G}, p >$ where $\mathcal{G} =< \mathbf{V}, \mathbf{E} >$ is a directed acyclic graph for which $\mathbf{V} = \mathbf{X} \cup \mathbf{Z} \cup \{Y\}$ and there is an edge from $V_i \in \mathbf{V}$ to $V_j \in \mathbf{V}$ only if V_i is a cause of V_j w.r.t. Pearl's do-notation (Pearl, 2009), and p is a probability distribution function over $\mathbf{V}$.

We now define the problem aforementioned problem, that we call the MaxCRD problem.

Definition 2. ***MaxCRD Problem.*** *Given a Bayesian Network* B*, output* $\top$ *if the rule* $\sigma^* = \arg\max_\sigma |e(\sigma)|$ *with the highest absolute effect has a non-zero effect.*

The following theorem proves that the MaxCRD problem is computationally difficult.

Theorem 2. *MaxCRD is NP-hard.*

Proof. We prove that MaxCRD is NP-hard by reducing from SAT. The proof is divided in two steps: first we show a polynomial-time reduction of an input of SAT to an input of MaxCRD, and then we show that solving MaxCRD on such input allows to derive a solution to SAT in time polynomial on the original instance.

We start by describing the reduction from SAT. Let $\psi(\mathbf{X})$ be a boolean formula over variables in $\mathbf{X}$. Let us define $\mathcal{G} =< \mathbf{V}, \mathbf{E} >$ with $\mathbf{V} = \mathbf{X} \cup \{Y\}$ and $\mathbf{E} = \{X_i \rightarrow Y | X_i \in \mathbf{X}\}$. Let us define each $X_i \sim \mathcal{B}(0.5)$ be a Bernoulli distribution with probability $p(X_i = 0) = p(X_i = 1) = 0.5$. Let Y take values in $\{0, 1\}$ and let $p(Y = 1 | X_1 = x'_1, ..., X_n = x'_n) = 1$ if and only if $\psi((x'_1, ..., x'_n)) = \top$ else $p(Y = 1 | X_1 = x'_1, ..., X_n = x'_n) = 0$[5]. We then define the BN $B =< \mathcal{G}, p >$ as the reduced input for MaxCRD.

We now prove that solving MaxCRD on the reduced input leads to solving SAT in polynomial time on the original instance by proving that (i) if MaxCRD(B) = $\top$ then $\psi(\mathbf{X}) = \top$ and (ii) if MaxCRD(B) = $\bot$ then we can build a polynomial-time algorithm that solves SAT.

Let us prove (i). If MaxCRD(B) = $\top$ then $\exists \sigma | e_{corr}(\sigma) \neq 0$ that is $p(Y = y | \sigma = \top) - p(Y = y | \sigma = \bot) \neq 0$. By construction, we have two cases: $y = 1$ or $y = 0$. If $y = 1$ then $\psi(\mathbf{X})$ is satisfiable by construction since at least one between $p(Y = 1 | \sigma = \top)$ and $p(Y = 1 | \sigma = \bot)$ is positive. (Note that $\sigma = \bot$ corresponds to all assignments of variables $\mathbf{X}$ for which rule σ is not satisfied, and $p(Y = 1 | \sigma = \bot) > 0$ if and only if at least one such assignment lead to $Y = 1$, that by definition implies that such assignment satisfies $\psi(\mathbf{X})$.) If $y = 0$ then we notice that the same rule evaluated on $y = 1$ has a non-zero effect given that $y = 1$ is $y = 0$'s complementary event therefore $p(Y = 0 | \sigma = \bot) = 1 - p(Y = 1 | \sigma = \bot)$ (and the same holds for $\sigma = \bot$).

Let us prove (ii). If MaxCRD(B) = $\bot$ then $\forall \sigma$ we have $p(Y = y | \sigma = \top) = p(Y = y | \sigma = \bot) = p(Y = y)$ that is the value of Y is independent on $\mathbf{X}$ assignments. This means that $\psi(\mathbf{X})$ is either a tautology or a contradiction[6] and by evaluating $\psi(\mathbf{X})$ on any assignment we can distinguish between the two cases. □

As stated before, in practice we do not have access to the exact probabilities and, therefore, to the exact effect $e(\sigma)$ for a rule σ. We are therefore interested in finding the rule with the largest positive *reliable* effect from an observational dataset, which we formalize in the problem below.

Definition 3. ***Max Reliable Positive CRD Problem.*** *Consider an observational dataset* $\mathcal{D}$ *on variables* $\mathbf{Z} \cup \mathbf{X} \cup \{Y\}$ *and a confidence level* $\alpha \in (0, 1)$*. Find the rule* σ^* *such that i)* $\hat{e}_{rel}(\sigma^*) > 0$ *and ii)* $\sigma^* = \arg\max_\sigma \hat{e}_{rel}(\sigma)$.

2 Proof of Theorem 1

ALLSTAR($\mathbf{X}$, $\mathbf{Z}$, y, ℓ, α, $G = (\mathbf{X}, E)$, k, t) outputs a set of rules with $FWER \leq \alpha$.

Proof. $[Sketch]$ Let us notice that each iteration of the for loop at line 6 considers an increasingly small subset of $\mathbf{X}$ and therefore the total amount N of candidate causal rules that may be evaluated by ALLSTAR (i.e. the total number of hypotheses tested in the worst scenario) is equal to the total number of rules that can be evaluated on the first iteration of the loop. In particular, the number of all the different rules of max length ℓ (i.e. N, line 1) is equivalent to the number of distinct connected subgraphs in G of length at most ℓ since ALLSTAR exploits G to expand a rule σ to a more specific $\sigma' \supset \sigma$ by adding a proposition $X_i = 1$ only if X_i is not already present in σ and it is connected to at least one treatment of σ.

We now prove that, by setting $\alpha_c = \alpha / N$ (line 1), ALLSTAR returns a false positive with probability at most α. Let us suppose that a false positive rule σ_{FP} (i.e. such that $e(\sigma_{FP}) \leq 0$) is returned in output by ALLSTAR. A necessary condition for this to happen is to add σ_{FP} to the top-k rules found (line 12) which in turn happens only if its estimated effect $\hat{e}_\sigma$ (calculated in line 9) is greater than 0 (line 12). By construction of the confidence intervals with confidence α_c, a rule with $e(\sigma_{FP}) \leq 0$ may have its estimated effect $\hat{e}_\sigma > 0$ with probability at most α_c. Since there are at most N rules under study, in the worst case the probability of having at least a false positive estimate is $N \times \alpha_c = \alpha$ which implies that the algorithm does not output any false positive with probability of at least $1 - \alpha$. □

3 ALLSTAR: Extended Description and Analysis

3.1 Algorithm Description

ALLSTAR starts by computing the total number of *candidate* rules of length at most ℓ (that is the number of connected subgraphs in G of length at most ℓ) and then calculates the correct threshold α_c for each confidence bound (see Section 2.2) using Bonferroni correction (line 1). The rule

[5] Note that the probability distribution function is fully specified since $p(Y = 0 | X_1 = x'_1, ..., X_n = x'_n) = 1 - p(Y = 1 | X_1 = x'_1, ..., X_n = x'_n)$.

[6] If not, then it would be possible to discover a rule with non-zero effect $\sigma : X_1 = x'_1 \wedge ... \wedge X_n = x'_n$ on all elements of $\mathbf{X}$. By construction, in fact, $p(Y = 1 | \sigma = \top) \in \{0, 1\}$ since it evaluates on just one element, and $p(Y = 1 | \sigma = \bot) \neq p(Y = 1 | \sigma = \top)$ otherwise the value of Y would be constant.

discovery is then performed in k iterations (line 6). In each iteration, a breadth-first search (BFS) of the lattice defined by set of all possible rules with at most ℓ alterations is performed by using a FIFO queue `Q` and its (standard) operations `enqueue` and `dequeue`. During the BFS, the best rule $\sigma_{\max}$, and its maximum reliable estimated effect $\hat{e}_{\max}$, discovered during the exploration are maintained. After the initialization of $\sigma_{\max}$ and $\hat{e}_{\max}$ (line 4), the queue `Q` is initialized by inserting the rules containing a single alteration (line 5). (Note that `ALLSTAR` can also consider the *absence* of an alteration as part of a rule (i.e. $X_i = 0$); for clarity's sake, this is not reported in Algorithm 1.) The BFS then proceeds by extracting the current rule σ (line 7) until `Q` is not empty (line 6). When a rule σ is extracted from `Q`, an upper bound to its reliable effect is computed with the function `computeRelATE`$(\sigma, y, \mathbf{Z}, \alpha_c)$. If such upper bound is greater than $\hat{e}_{\max}$ (line 8) then the (exact) reliable effect estimate $\hat{e}_\sigma$ of σ is computed (line 9), and the values $\hat{e}_{\max}, \sigma_{\max}$ are updated if $\hat{e}_\sigma > \hat{e}_{\max}$ (line 10). Then, the rules that are obtained by expanding σ, obtained with the function `expand`(σ, G, ℓ), are added to the queue (lines 11-11). `expand`(σ, G, ℓ) returns all rules (with at most ℓ alterations) that are obtained by adding to σ one alteration that must be connected in G to at least one alteration of σ. When the BFS completes, the best rule $\sigma_{\max}$ is added to the output set if its estimated reliable effect is positive (line 12), and the set $\mathbf{X}$ of alterations is updated (line 13) to avoid discovering highly-overlapping, redundant, rules (see below). At the end, the set of at most top-k rules is reported in output (line 16).

Algorithm 2: `calculateRulesNumber`

Input: Graph $G = (\mathbf{V}, \mathbf{E})$, maximum rule length ℓ
Output: Number N of connected subgraphs of length at most ℓ between elements in G

```
P ← ∅;
Q ← ∅;
for X ∈ V do
    P ← P ∪ {X};
    Q ← Q ∪ {X};
for i ← 1 to ℓ − 1 do
    L ← ∅;
    for q ∈ Q do
        for X ∈ q do
            for e ∈ E do
                if X ∈ e & e \ {X} ∉ q then
                    L ← L ∪ {q ∪ {e \ {X}}}
    Remove duplicates from L;
    P ← P ∪ {L};
    Q ← L;
return size(P);
```

`ALLSTAR` exploits three subroutines `calculateRulesNumber`, `upperBoundRelATE`, and `computeRelATE` that will be briefly explained in the following (and whose Python code is available online): **`calculateRulesNumber`** takes as input a graph G and the maximum rule length ℓ and outputs the number of connected subgraphs of length at most ℓ between elements in G. It is used to calculate the total number of possible rules under study, which is the amount of test performed in the worst case, and the pseudocode is described in Algorithm 2.
`upperBoundRelATE` takes as an input a rule σ, the value y for target Y, a set of confounders $\mathbf{Z}$, and a threshold α_c corrected for multiple hypotheses testing, and it outputs the tight optimistic upper bound to the effect for the rule proposed by Budhathoki *et al.* (2021). It is used by the branch-and-bound algorithm for deciding whether to compute study a specific branch (i.e. all children of a specific rule) or to avoid the computation because the best solution found in such branch would never improve the current best solution (i.e., the incumbent) $\hat{e}_{max}$.
More specifically, let us consider a rule σ and a more specific rule $\sigma' = \sigma \wedge \pi_j$. Let us define the quantity $\tilde{\tau}_{\sigma'}(\sigma, \mathbf{z})$ on the elements for which $\mathbf{Z} = \mathbf{z}$ holds as

$$\tilde{\tau}_{\sigma'}(\sigma, \mathbf{z}) = \max_{a'_\sigma \in \{0,1,\ldots,a_\sigma\}} \frac{a'_\sigma + 1}{a'_\sigma + 2} - \frac{n_1 - a'_\sigma + 1}{n - a'_\sigma + 2} + - \frac{\beta(\alpha_c)}{2\sqrt{a'_\sigma + 2}} - \frac{\beta(\alpha_c)}{2\sqrt{n - a'_\sigma + 2}}$$

where $\beta(\alpha_c)$ is the $1 - \alpha_c/2$ quartile of the standard normal distribution, n is the number of instances taken into account (i.e. with $\mathbf{Z} = \mathbf{z}$), n_1 of which have $Y = y$, and a_σ is the number of instances for which σ holds, $\mathbf{Z} = \mathbf{z}$ and $Y = y$. The upper bound is then defined as

$$U(\sigma') = \sum_{\mathbf{z}} (\tilde{\tau}_{\sigma'}(\sigma, \mathbf{z}) \hat{p}(\mathbf{Z} = \mathbf{z}))$$

where $\hat{p}(\mathbf{Z} = \mathbf{z})$ is the empirical probability of $\mathbf{Z}$ taking value $\mathbf{z}$. Differently from Budhathoki *et al.* (2021) our bound uses a confidence level $\alpha_c = \alpha/N$, where N is the total number of rules considered by the algorithm, to account for the multiple hypothesis testing problem.
`computeRelATE` takes as an input a rule σ, the value y for target Y, a set of confounders $\mathbf{Z}$, and a threshold α_c and calculates the reliable effect of the rule $\hat{e}_{rel}(\sigma)$ as described in Section 2.2. More specifically, let us define $\hat{p}(Y = y | \sigma = \top) = \frac{n_{Y=y,\sigma=\top}}{n_{\sigma=\top}}$ where $n_{\sigma=\top}$ is the number of instances for which $\sigma = \top$ (i.e, σ is true), and $n_{Y=y,\sigma=\top}$ is the number of instances for which $Y = y$ and $\sigma = \top$. Analogously we have $\hat{p}(Y = y | \sigma = \perp) = \frac{n_{Y=y,\sigma=\perp}}{n_{\sigma=\perp}}$. In extreme cases (e.g. $\sigma = \perp$ for all instances) such quantities are ill-defined, therefore the Laplace correction is applied to the estimated conditional probability, which becomes $\hat{p}_c(Y = y | \sigma = \top) = \frac{n_{Y=y,\sigma=\top}+1}{n_{\sigma=\top}+2}$. The returned value $\hat{e}^y_{rel}(\sigma)$ is then defined as (for more details on the idea, see Section 5 in Supplemental material)

$$\hat{e}^y_{rel}(\sigma) = \sum_{\mathbf{z}} \Bigg[\Bigg(\hat{p}_c(Y = y | \mathbf{Z} = \mathbf{z}, \sigma = \top) + - \hat{p}_c(Y = y | \mathbf{Z} = \mathbf{z}, \sigma = \perp) + - \frac{\beta(\alpha_c)}{2\sqrt{n_{\mathbf{Z}=\mathbf{z},\sigma=\top}}} - \frac{\beta(\alpha_c)}{2\sqrt{n_{\mathbf{Z}=\mathbf{z},\sigma=\perp}}} \Bigg) \hat{p}(\mathbf{Z} = \mathbf{z}) \Bigg].$$

3.2 Stability Analysis

We experimentally assessed the stability of `ALLSTAR` results with respect to the user-defined parameters ℓ, t, and $G = (\mathbf{X}, E)$.

In the first experiment, we run `ALLSTAR` on the datasets of the synthetic experiment of Sec. 5 in the Supplemental Material, with ℓ taking values from 3 to 7, and $t = 0.05$. Figure 3 shows the average effect returned by `ALLSTAR` (solid lines) for the three implanted rules by varying ℓ, as well as their theoretical value (dashed lines). Results have been averaged for all datasets of 1000 samples, and their variability across the runs is negligible. As expected, the rule effect returned by `ALLSTAR` decreases as the rule length increases because the number of hypotheses to test increases and therefore the Bonferroni correction becomes stricter. Moreover, despite increasing ℓ allows `ALLSTAR` to evaluate more rules, our algorithm did not return any false positive.

We then analyzed results variability w.r.t. changes of t by running `ALLSTAR` on the same setup of the previous experiment, and setting t

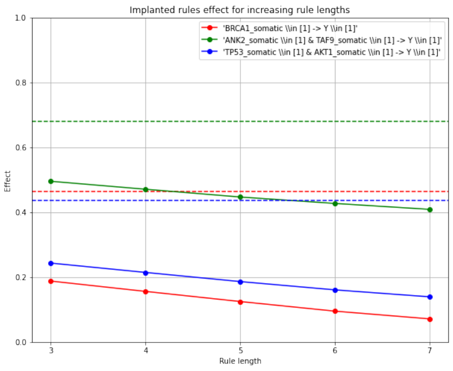

Fig. 3: Average effect returned by ALLSTAR (solid lines) and theoretical value (dashed lines) for the 3 implanted rules of the last syntetic experiment (see Sec. 5 in the Supplemental Material). Results have been averaged on datasets with 1000 samples, and the variability in each run results is negligible.

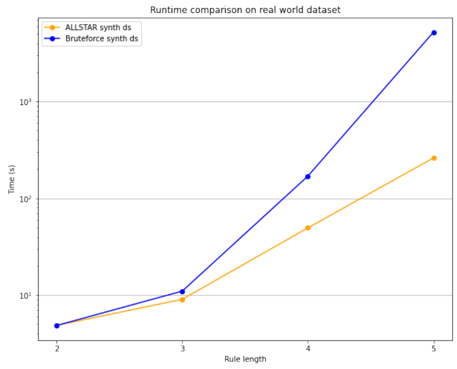

Fig. 4: Average runtime comparison between ALLSTAR and a brute-force algorithm on 10 synthetic datasets over different rule lengths ℓ. Y-axis is logarithmically scaled, and variability across runs with the same ℓ is negligible (and therefore not plotted).

to $0.01, 0.025, 0.05, 0.075$, and 0.1. We finally set $k = 4$ to assess the ability of ALLSTAR to avoid returning duplicated rules. ALLSTAR returned duplicate rules consistently among all the runs for $t = 0.01$, among 6 of 10 runs for $t = 0.025$, and did not return any duplicated rule for all the other values of t tested. This is an expected behavior in this data generative scenario since each rule differs from its clone on 2.5% of samples on average (see equations in Sec. 5 in the Supplemental Material).

Lastly, we evaluated the impact of using a high confidence subnetwork of Reactome's Protein-Protein Interaction (PPI) (see Section 4.1), as input knowledge. Conveniently, the original PPI from Reactome is featured with a score ($s \in [0, 1]$) for each pair of genes, representing the confidence of their edge in the interaction network. To build the experiment, we removed every pair with a score lower than 1, thus keeping only high confidence links, and run ALLSTAR with the same data inputs and parameters as described in Section 4.3, with the exception of the PPI. We then compared the results obtained running our algorithm with the two PPIs. Keeping Table 1 as reference, a total of 7 rules out of 26 (27% of the reference), and specifically, rules b, h, l, m, r, u, and v, were not retrieved in this analysis. These results show that most of the rules found by ALLSTAR including lower confidence interactions, are still reported using only high-confidence interactions. Moreover, the excluded rules r and u were not extensively characterized by our oncologist due to lack of literature support. Conversely, rules b, h, l, m, and v were labeled as potentially novel discoveries: as motivated in Section 4.3, these rules refer to proteins whose impact on breast cancer is debated. Even if their role in breast cancer physiology is not specifically supported by sufficient literature, the underlying biological mechanisms are explainable, either because of their genetic properties and functionalities, or the existence of an analogous biological process in other cancer types. Overall, these results show that ALLSTAR can focus on well-characterized mechanisms by including only high-confidence interactions, but also that ALLSTAR can be used in to pinpoint potential novel discoveries by including lower-confidence interactions.

3.3 Computational Performances

We finally compared the computational performances of ALLSTAR against a brute-force algorithm that exploits G to select the candidate rules to study, but calculates them all without exploiting the branch-and-bound. In this experiment we created 10 synthetic datasets with 1000 samples from the following distributions

$$
\begin{aligned}
X_1 &\sim \mathcal{B}(0.15) \\
E_i &\sim \mathcal{B}(0.1), 1 \le i \le 600 \\
Y &\sim X_1 \vee \mathcal{B}(0.05)
\end{aligned}
$$

and we searched for the rule with the highest effect ($k = 1$) by setting the target value $Y = 1$. We run both algorithms on 60 cores of our cluster and we tracked the runtimes without considering the time required to calculate the Bonferroni correction (i.e. function `calculateRulesNumber` of ALLSTAR) as our focus is to compare the performances of the two rule discovery approaches only[7]. Figure 4 compares the average runtimes in seconds of both approaches (y axis is log-scaled) over increasing maximum rule lengths ℓ. As expected, ALLSTAR is faster than the brute force approach due to the speedup given by its branch-and-bound, and such difference increases with the number of rules under study, therefore it increases monotonically with ℓ. As a reference, the brute force algorithm is more than 3 times slower than ALLSTAR when discovering rules setting $\ell = 4$, and nearly 20 times slower for $\ell = 5$.

4 Improved Bound Description

Consider a rule $\sigma = \pi_1 \wedge \ldots \wedge \pi_i$ and a more specific one $\sigma' = \sigma \wedge \pi_j$. Budhathoki *et al.* (2021) defined the upper bound $\tilde{\tau}_{\sigma'}(\sigma, \mathbf{z})$ to the reliable effect estimate $\hat{e}_{rel}(\sigma')$ of σ' as a function of the number of instances n (in the $\mathbf{Z}$ strata), the number n_1 of instances with $Y = y$, and the number a_σ of instances for which σ holds and $Y = y$, as

$$
\tilde{\tau}_{\sigma'}(\sigma, \mathbf{z}) = \max_{a'_\sigma \in \{0,1,\ldots,a_\sigma\}} \frac{a'_\sigma + 1}{a'_\sigma + 2} - \frac{n_1 - a'_\sigma + 1}{n - a'_\sigma + 2} + \\
- \frac{\beta(\alpha)}{2\sqrt{a'_\sigma + 2}} - \frac{\beta(\alpha)}{2\sqrt{n - a'_\sigma + 2}}
$$

which upper bounds the effect of σ' by exploiting the fact that a_σ will upper bound the number $a_{\sigma'}$ of instances for which the σ' holds and $Y = y$, given that σ' is more specific than σ. We argue that σ' not only is more specific than σ, but also than every rule in the set $\Omega_p = \{\sigma' \setminus \{\wedge \pi_k\} | \forall \pi_k \in \sigma'\}$ of all possible rules chosen from σ' removing the proposition π_k. The proposed estimator must hold for each rule in Ω_p

[7] We remind that such procedure would be a prerequisite for both algorithms, therefore it would just add a bias term to both runtimes under analysis.

therefore we propose a tighter optimistic estimator that considers $a_{min} = \min_{\sigma_j \in \Omega_p} a_{\sigma_j}$ as

$$\bar{\tau}_{\sigma'}(\sigma, \mathbf{z}) = \max_{a'_{\Omega_p} \in \{0,1,\dots,a_{min}\}} \frac{a'_{\Omega_p}+1}{a'_{\Omega_p}+2} - \frac{n_1 - a'_{\Omega_p}+1}{n - a'_{\Omega_p}+2} + \\ - \frac{\beta(\alpha)}{2\sqrt{a'_{\Omega_p}+2}} - \frac{\beta(\alpha)}{2\sqrt{n - a'_{\Omega_p}+2}}$$

Notice that if a rule $\sigma_{rem} \in \Omega_p$ has been pruned by the breadth-first branch and bound algorithm, then we can set $\bar{\tau}_{\sigma'}(\sigma, \mathbf{z}) = -\infty$ since the condition in line 8 does not hold for any such σ', given that it is more specific than σ_{rem}.

5 Synthetic Experiments

Probability and Rule Effect Estimation

The estimation of probabilities from data is challenging when sample sizes are small, as the estimates obtained with naïve empirical estimators have high variance. As a consequence, rules discovered by data using such naïve empirical estimators have effects whose estimates are far from their true effects. To mitigate this phenomenon, which may lead to overfitting, Budhathoki *et al.* (2021) proposes a *reliable* estimator for the effect of causal rules.

By considering all samples such that $\sigma = \top$ (resp. $\sigma = \perp$), the value y is a binomial distribution with success probability $\hat{p}(Y = y|\sigma = \top)$ (defined in Section 3.1 of the Supplemental Material). For a given confidence level $\alpha \in (0,1)$, by defining $\beta(\alpha)$ as the $1-\alpha/2$ quantile of a standard normal distribution, the confidence bound for $\hat{p}_c(Y = y|\sigma = \top)$ proposed by Budhathoki *et al.* (2021) is then

$$\left[\hat{p}_c(Y = y|\sigma = \top) - \frac{\beta(\alpha)}{2\sqrt{n_{\sigma=\top}}}, \hat{p}_c(Y = y|\sigma = \top) + \frac{\beta(\alpha)}{2\sqrt{n_{\sigma=\top}}}\right].$$

Such bound allows us to compute the effect of **reliable causal rules**, defined as the lower bound of the effect of causal rules. That is, the estimated reliable effect $\hat{e}^y_{rel}(\sigma)$ of a causal rule σ on Y taking value y with confidence α is defined as:

$$\hat{e}^y_{rel}(\sigma, \alpha) = \hat{p}_c(Y = y|do(Q_\sigma)) - \hat{p}_c(Y = y|do(Q_{\bar{\sigma}})) + \\ - \frac{\beta(\alpha)}{2\sqrt{n_{\sigma=\top}}} - \frac{\beta(\alpha)}{2\sqrt{n_{\sigma=\perp}}}.$$

where Q_σ is a *stochastic policy* i.e., a probability distribution over the interventions (see Budhathoki *et al.* (2021) for more details), which combines *atomic interventions* $do(X = x)$ (Pearl, 2009), i.e. changes the value of the variable X_i to x_i while keeping the values of all the other variables fixed.

Our method applies Bonferroni correction to $\hat{e}^y_{rel}(\sigma)$ by using a threshold $\alpha_c = \alpha/N$ where N is the total number of rules under study.

Preliminary Synthetic Experiment Extension

In the preliminary synthetic experiment, ALLSTAR was compared to standard correlational approaches, proving its ability to retrieve different results among the top rules (i.e. the ones that rank higher in terms of reliable effect, odds-ratio and p-value). For completeness' sake, we took a step further and replicated the same experiment in a real-world example that includes confounders. We argue that our findings in the synthetic setting are exacerbated in the real-world scenario. We chose one of the breast cancer-related datasets we described in Sec. 4.3. Specifically, we selected the one with the 300 most frequent somatic mutations, the 300 most frequent LOHs, and the profiles of 22 frequently hypermethylated genes, as $\mathbf{X}$, and the Triple-Negative binary molecular classification, as target Y. We then ran both ALLSTAR and a python implementation of the Cochran–Mantel–Haenszel test (CMH), ranking reliable effects and p-values for rules built on every combination of one confounder, two alterations and the outcome. The scatter plot describing the two rankings' comparison for the first 1000 rules sorted by reliable effect, is shown in Fig. 5. It seems clear that a considerable amount of rules ranked among the top-1000 in the effect ranking, when assessed using the correlation-based method CMH, are placed well beyond the 100000th position. Additionally, the p-value rankings compressed to the bottom of the plot are actually all valued 1. CMH is not able to differentiate all these rules, giving them a p-value of zero, which hints at their possible significance, but fails at prioritizing the combinations of genes that may be relevant to the Triple-Negative phenotype.

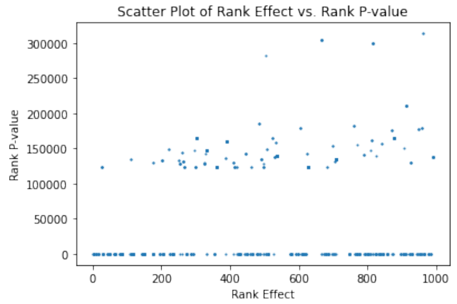

Fig. 5: Comparison of the rankings in terms of reliable effect (ALLSTAR output, x-axis) and p-value (CMH test output, y-axis) for real-world data. Each dot corresponds to one of the top-1000 rules ranked by reliable effect.

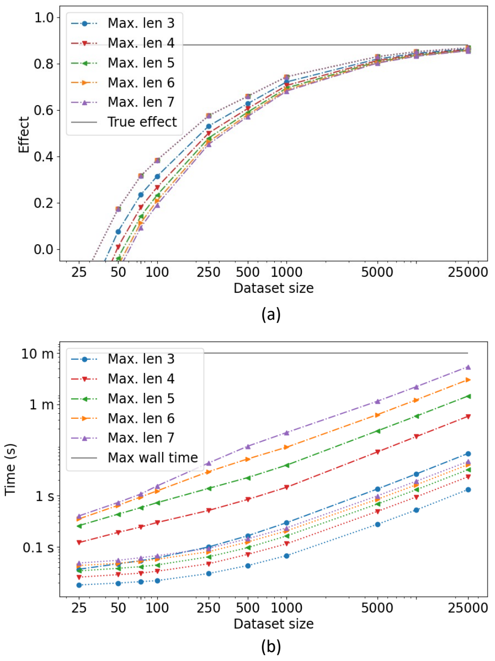

Fig. 6: Mean planted reliable rule effect (a) and mean runtimes (b) over multiple dataset sizes on 10 runs. In each plot, the dotted lines represent ALLSTAR results passing a protein-protein interaction G in input, and dash-dotted lines represent the approach with a fully connected graph (i.e., no prior knowledge).

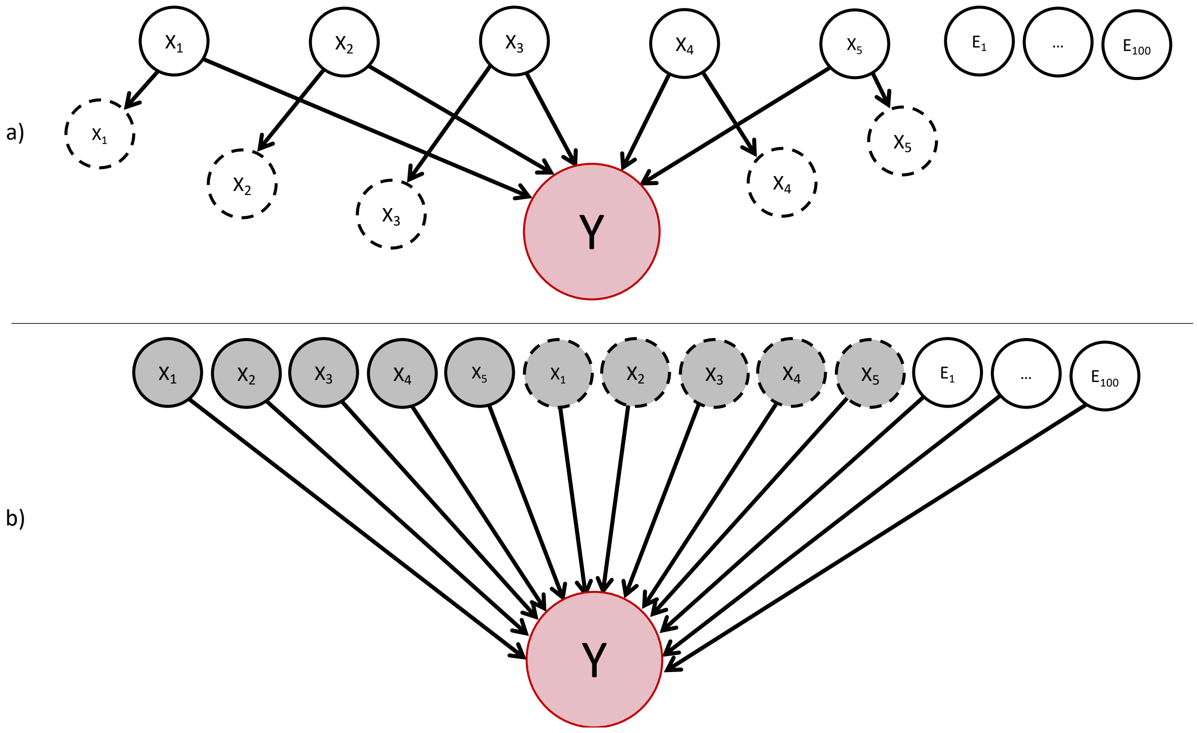

Fig. 7: Data generative BN (a) and assumed graph (b) of last synthetic experiment. In the second plot, clone variables (i.e. those which definition depends on other variables) are shown with a dashed border, and variables that output rules with a positive effect by ALLSTAR without cleaning procedure are represented in grey.

Comments on Second Synthetic Experiment

Figure 6 shows the results obtained passing G in input (dotted line) and the results obtained when no prior knowledge on gene interaction is considered (dash-dotted line), obtained by passing a fully connected graph in input to ALLSTAR. In particular, we considered both the effect estimation of the implanted rule and the runtime.

Comments on Last Synthetic Experiment

In the last synthetic experiment, ALLSTAR without cleaning procedure reported spurious associations (and not true causations) due to a failure of Assumption 3 for the admissible causal structure (see Sec. 2.1). In particular, the graph of the data generative BN was the one shown in Figure 7(a), while the one assumed by ALLSTAR was 7(b). The main difference between the two BNs are the d-separations between $X_i, 1 \leq i \leq 5$ and their clones $X_{i(clone)}$. In particular, X_i always blocks spurious correlation paths (more on this and *d-separation* in Pearl (2009)) from $X_{i(clone)}$ in 7(a) but not in 7(b), therefore if we (incorrectly) assume the underlying graph to be as the latter, in order to still have correct results we should be able to have some other heuristic mechanism (the threshold-based cleaning procedure) that removes the clones in order not to return them. Let us also notice that another difference between 7(a) and 7(b) relies on the links between the external variables ($E_i, 1 \leq i \leq 100$) and Y. Such links imply some form of (possible) dependence whose strength is defined by the probability distribution functions inferred by the observational dataset. ALLSTAR however, is able to confidently ignore such spurious correlations due to the use of the *reliable* effect estimator and its ability to deal with multiple hypoteses testing (more on that on Theorem 1 proof). Analysis of relaxation of the other assumptions (and the consequent development of new methods) is still an open research task, for which we point the reader to the discussion on Budhathoki *et al.* (2021). Equations for sampling data from the graph of Figure 7(a) follows:

$$\begin{aligned}
X_1 &\sim \mathcal{B}(0.5) \\
X_2 &\sim \mathcal{B}(0.4) \\
X_3 &\sim \mathcal{B}(0.7) \\
X_4 &\sim \mathcal{B}(0.65) \\
X_5 &\sim \mathcal{B}(0.15) \\
X_{i(clone)} &\sim X_i \oplus \mathcal{B}(0.025) \\
E_i &\sim \mathcal{B}(0.5) \\
Y &\sim (X_1 \wedge X_2) \vee (X_3 \wedge X_4) \vee X_5
\end{aligned}$$

6 Comparison with naïve greedy selection

Lastly, we compare ALLSTAR with a naïve greedy selection of the top-k rules by effect, without considering any cleanup threshold t. Let us consider the scenario of the Second Synthetic experiment, in which we consider datasets with a rule σ implanted composed of 7 elements. By considering rules length $\ell \in \{2, 3, 4, 5\}$, all the elements of the implanted rule are returned by ALLSTAR by just setting $k = \lceil \frac{7}{\ell} \rceil$. In other words, all elements of σ are in the top-4 rules returned by ALLSTAR for $\ell = 2$, in the top-3 for $\ell = 3$, and in the top-2 for $\ell \in \{4, 5\}$. A greedy algorithm that ranks per effect all the rules of length ℓ and selects the top-k without exploiting a cleanup threshold, however, was never able to discover all the important genes in σ and always returned rules with repeated genes. Such results are sound w.r.t. different dataset sizes (as we tested datasets with 100, 1000, and 10000 samples) and statistical noises (as we tested 10 datasets per sample size).
